# An Individualized Prognostic Model in Patients with Locoregionally Advanced Nasopharyngeal Carcinoma Based on Serum Metabolomic Profiling

**DOI:** 10.3390/life13051167

**Published:** 2023-05-11

**Authors:** Jiayu Zhou, Yishu Deng, Yingying Huang, Zhiyi Wang, Zejiang Zhan, Xun Cao, Zhuochen Cai, Ying Deng, Lulu Zhang, Haoyang Huang, Chaofeng Li, Xing Lv

**Affiliations:** 1 State Key Laboratory of Oncology in South China, Guangdong Key Laboratory of Nasopharyngeal Carcinoma Diagnosis and Therapy, Sun Yat-sen University Cancer Center, Guangzhou 510060, China; zhoujy@sysucc.org.cn (J.Z.); dengys@sysucc.org.cn (Y.D.); huangyy4@sysucc.org.cn (Y.H.); zhanzj@sysucc.org.cn (Z.Z.); caoxun@sysucc.org.cn (X.C.); caizc@sysucc.org.cn (Z.C.); dengying1@sysucc.org.cn (Y.D.); zhangll3@sysucc.org.cn (L.Z.); huanghy2@sysucc.org.cn (H.H.); 2 Department of Nasopharyngeal Carcinoma, Sun Yat-sen University Cancer Center, Guangzhou 510060, China; 3 Department of Information, Sun Yat-sen University Cancer Center, Guangzhou 510060, China; 4 School of Electronics and Information Technology (School of Microelectronics), Sun Yat-sen University, Guangzhou 510275, China; 5 The First School of Clinical Medicine, Southern Medical University, No. 1023, South Shatai Road, Baiyun District, Guangzhou 510515, China; wangzy_gz@163.com; 6 Department of Critical Care Medicine, Sun Yat-sen University Cancer Center, Guangzhou 510060, China

**Keywords:** locally advanced nasopharyngeal carcinoma, metabolomics, biomarker, prognostic model

## Abstract

Purpose: This study aims to evaluate the value of a serum metabolomics-based metabolic signature for locoregionally advanced nasopharyngeal carcinoma (LA-NPC) patients, thereby assisting clinical decisions. Methods: In this retrospective study, a total of 320 LA-NPC patients were randomly divided into a training set (ca. 70%; *n* = 224) and a validation set (ca. 30%; *n* = 96). Serum samples were analyzed using widely targeted metabolomics. Univariate and multivariate Cox regression analyses were used to identify candidate metabolites related to progression-free survival (PFS). Patients were categorized into high-risk and low-risk groups based on the median metabolic risk score (Met score), and the PFS difference between the two groups was compared using Kaplan–Meier curves. The predictive performance of the metabolic signature was evaluated using the concordance index (C-index) and the time-dependent receiver operating characteristic (ROC), and a comprehensive nomogram was constructed using the Met score and other clinical factors. Results: Nine metabolites were screened to build the metabolic signature and generate the Met score, which effectively separated patients into low- and high-risk groups. The C-index in the training and validation sets was 0.71 and 0.73, respectively. The 5-year PFS was 53.7% (95% CI, 45.12–63.86) in the high-risk group and 83.0% (95%CI, 76.31–90.26) in the low-risk group. During the construction of the nomogram, Met score, clinical stage, pre-treatment EBV DNA level, and gender were identified as independent prognostic factors for PFS. The predictive performance of the comprehensive model was better than that of the traditional model. Conclusion: The metabolic signature developed through serum metabolomics is a reliable prognostic indicator of PFS in LA-NPC patients and has important clinical significance.

## 1. Introduction

Nasopharyngeal carcinoma (NPC) is a malignant epithelial tumor with unique geographic distribution characteristics; it is primarily found in Southern China and Southeast Asia [1]. Due to its insidious onset and non-specific symptoms in early stages [2], nearly 75% of the patients present with locoregionally advanced stages at initial diagnosis [3]. Patients with advanced NPC have a poorer prognosis, with approximately 30% of patients suffering treatment failure [4]. Prognostic prediction and therapeutic decisions in NPC are dictated primarily by the tumor, lymph node, and metastasis (TNM) staging system, which is based on anatomic factors [5]. However, this staging system has some limitations because clinical outcomes can vary significantly among patients at the same stage [6]. Hence, TNM staging seems insufficient to reflect individual heterogeneity between patients and accurately predict patient prognosis. Besides, hypoxia, proliferation, cancer stem cells, and radiotherapy resistance are major contributors to tumor heterogeneity, which in turn leads to diverse treatment responses. Hypoxia promotes cancer cell proliferation, migration, and radiotherapy resistance, while inhibiting apoptosis and immune responses [7]. Proliferation results in diverse growth rates and response to treatments [8]. Cancer stem cells have the ability to self-renew and produce different types of cancer cells, and are involved in tumor development, recurrence, and drug resistance [9]. Radiotherapy resistance refers to the decreased sensitivity or increased tolerance of tumors to radiation [10]. These factors, in various combinations and interactions, lead to significant tumor heterogeneity and require personalized treatment strategies. In tumor research, biomarker studies encompass multiple aspects such as tumor-related genes, proteins, metabolites, and more. Analyzing the expression levels and genetic variations of these biomarkers can lead to a better understanding of tumor heterogeneity, enabling the identification of patients with an increased risk of disease progression, prioritization of therapeutic interventions, and improvement of clinical outcomes [11]. Previous studies have demonstrated that some biomarkers can predict survival outcomes for locoregionally advanced nasopharyngeal carcinoma (LA-NPC), such as Epstein–Barr virus DNA (EBV DNA) concentrations [12], some inflammatory markers [13,14], and radiomics features of magnetic resonance imaging (MRI) or positron emission tomography/computed tomography (PET/CT) [15,16,17]. Nevertheless, the clinical utility of these factors is restricted and unclear [18]. First, the lack of standardization of image acquisition protocols and analysis methods can result in significant inter-study variability and hinder the generalization of the findings. Second, the limited interpretability of deep learning models makes it difficult to understand their internal mechanisms, which may lead to unreliable results. Third, the potential confounding effects of various clinical factors, such as treatment history and tumor stage, may confound the association between radiomics/deep learning features and patient outcomes [19,20]. In addition, as a prognostic marker, EBV DNA has certain limitations in its specificity and sensitivity, and the results of different laboratories vary greatly [21]. Thus, novel and powerful biomarkers that reflect tumor heterogeneity are urgently needed to identify patients at risk for poor prognosis.

Metabolomics, an emerging and promising field, has great potential for predicting the occurrence and development of tumors [22,23]. Compared with normal cells, metabolic activities are altered in cancer cells in many ways to maintain continuous proliferation and malignant properties [24]. These metabolic alterations are commonly observed in many types of cancer, and reprogrammed metabolism is regarded as a hallmark of cancer [23,25]. With numerous advancements in high-throughput metabolomics technologies, compared to traditional metabolomics technologies in the past, it is more accurate to screen and identify the differential metabolites that correlate with the disease phenotype [26]. Several studies have identified and validated the close association between metabolic characteristics and the occurrence and progression of various types of cancer [27,28]. However, the metabolomics for NPC is still in its infancy. Previous studies mainly focused on the early diagnosis of NPC [29,30,31], and there were few studies on the relationship between the prognosis of nasopharyngeal carcinoma and metabolomic features. Therefore, additional research in this area is still needed. Besides, most current metabolomic studies of nasopharyngeal carcinoma were analyzed using the untargeted metabolomics, which has certain limitations in identifying metabolites. Notably, widely targeted metabolomics based on multiple reaction monitoring, a novel metabolomics technology, subtly integrates the advantages of non-targeted and targeted metabolomics [32]. This high-throughput technology can not only detect hundreds of metabolites in batches but also shows higher sensitivity and accuracy in the identification and quantification of metabolites [33].

In this study, serum widely targeted metabolomic profiling based on Metabolon ultra-performance liquid chromatography–tandem mass spectrometry (UPLC–MS/MS) was conducted in 320 individuals with LA-NPC before treatment. We sought to develop a metabolic signature for predicting the prognosis of patients with LA-NPC and build a potential prognostic model based on metabolomics for predicting progress-free survival (PFS). We hope that our research will map the metabolomic landscape of LA-NPC and provide a new biomarker for tailoring treatment decisions.

## 2. Materials and Methods

### 2.1. Study Design and Clinical Specimens

**Figure 1:**
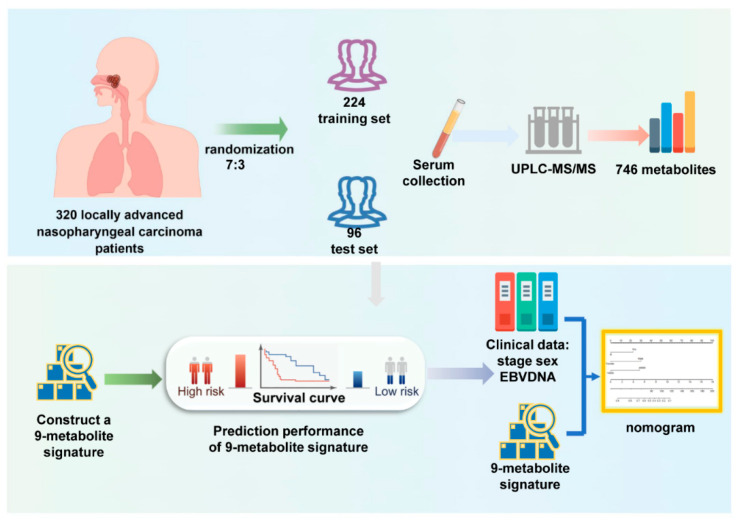
Workflow of the study design.

The overall study design and workflow are presented in Figure 1. A total of 320 patients with LA-NPC (stage III to IVa) treated at Sun Yat-sen University Cancer Center between 2013 and 2016 were enrolled in this study. The study population was divided randomly into a training set (224 cases, 70%) and a validation set (96 cases, 30%) aiming at a ratio of 7:3. According to the eighth edition of the American Joint Committee on Cancer (AJCC) Staging Manual, two radiologists reevaluated all patients by reassessing baseline MRI scans and resolved discrepancies by consensus. Complete baseline clinical information and follow-up data were available for these patients. All patients underwent a complete physical examination, MRI of the nasopharynx and neck, bone scan, fiberoptic nasopharyngoscopy, and complete blood sampling, including routine blood tests, biochemical characteristics, and EBV-DNA copies before treatment. Individuals with impaired heart, lung, liver, or kidney function and those with a history of malignant tumors were excluded from this study. All patients underwent platinum-based chemotherapy and intensity-modulated radiotherapy (IMRT). The Research Ethics Committee of the Sun Yat-sen University Cancer Center approved this study (B2022-429-01).

### 2.2. Clinical Endpoints and Follow-Up

The primary endpoint was 5-year PFS, and the secondary endpoints were overall survival (OS), 5-year distant metastasis-free survival (DMFS), and 5-year local-regional recurrence-free survival (LRFS). PFS was defined as the time from the initial diagnosis until relapse, progression, or death due to any cause, whichever occurred first. OS was defined as the time from diagnosis date to death from any cause. DMFS was defined as the time from diagnosis to data of distant recurrence, and LRFS was defined as the time from diagnosis to local or regional recurrence.

Patients underwent routine imaging every 3 months for the first 2 years and every 6 months for years 3–5. The follow-up duration was defined as the interval from diagnosis to death or the last follow-up (31 December 2020). All local and regional recurrences were confirmed by radiological examination and pathology. The diagnosis of distant metastases was based mainly on imaging methods, such as MRI, CT, or PET/CT.

### 2.3. Blood Sample Collection

To minimize the impact of food and nutrition on the serum metabolome and laboratory results, peripheral blood samples were collected in the morning under fasting conditions before the initial treatment. The blood samples were immediately centrifuged at 1000× *g* at 4 °C for 10 min to isolate serum, which was stored at −80 °C until assayed. Baseline routine hematology and biochemical assays were performed at admission using a fully automated hematology analyzer Sysmex XE-5000 (Sysmex, Kobe, Japan) and an automated immunoturbidimetric analyzer 7600–020 (Hitachi High-Technologies, Tokyo, Japan). The circulating EBV-DNA levels in the blood were detected using quantitative real-time polymerase chain reaction (RQ-PCR).

### 2.4. Widely Targeted Metabolite Profiling

We applied a pseudotargeted metabolomics strategy based on UPLC–MS/MS. The sample orders were randomized, and all samples were measured in a single batch. Serum samples were thawed on ice, vortexed for 10 s, and mixed well. We then added 300 μL of extracting solution, which consisted of internal standards and 20% methanol/acetonitrile, to 50 μL of serum. The mixture was stirred for 3 min and centrifuged at 12,000 rpm for 10 min at 4 °C. The supernatant was transferred to a new tube and centrifuged at 12,000 rpm for 5 min at 4 °C. The supernatant was then placed at −20 °C for 30 min before being centrifuged at 12,000× *g* for 5 min at 4 °C. Finally, 150 μL of supernatant was removed from a liquid chromatography (LC) injection bottle, and the injection volume was 2 μL for on-board analysis using LC-MS.

In the training and validation sets, widely targeted metabolite profiling of samples was performed using the self-built Metware database (MWDB) developed by MetWare Biotechnology Co. (Wuhan, China). In total, 746 endogenous water-soluble metabolites were detected using an UPLC–MS/MS system (ExionLC AD coupled to a QTRAP spectrometer). The specific information of all metabolites is given in Table S1. In order to facilitate the observation of changes in the relative content of metabolites, we used standardization processing (unit variance scaling, UV scaling) for the original relative content of differential metabolites identified by the screening criteria, and we then drew the heatmap. The result is shown in Figure S1. For additional details on the widely targeted metabolomics analysis and quality control analysis, see the experimental conditions in Supplementary Materials and Figure S2.

### 2.5. Metabolites Selection and Metabolomics Signature Building

All the samples were randomly divided into training and validation sets at a ratio of 7:3. The training set was used to calculate the risk score (Met score) and establish a prognostic model, whereas the validation set was used to verify the efficiency of the model. We used the R language to standardize the metabolome data (746 metabolites) with the Z-score method and screened out 27 metabolites associated with PFS by univariate Cox regression (false discovery rate [FDR] < 0.05) (Table S2). We used multi-factor Cox regression based on the screened metabolites to build a prognostic model, and we calculated the model’s Akaike information criterion (AIC) value using forward-backward stepwise regression. The smaller the AIC value of the model, the better the model fit the data. Finally, we obtained a metabolically related risk regression model named the 9-metabolite signature.

### 2.6. Prognostic Validation of 9-Metabolite Signature

We calculated the Met score for all patients with Cox regression coefficients of each metabolite in the 9-metabolite signature. Kaplan–Meier (KM) survival analysis was used to verify survival differences in both sets (log-rank test). The patients were divided into high- and low-risk groups using the median Met score. Then, time-dependent receiver operating characteristic (ROC) analysis and the concordance index (C-index) were applied to assess the predictive power of the 9-metabolite signature for PFS. At the same time, we also evaluated the prediction performance of the 9-metabolite signature in the secondary endpoints including 5-year OS, 5-year DMFS, and 5-year LRFS.

### 2.7. Performance of the Nomogram Based on Metabolomics and Traditional Clinical Factors

The prognostic value of the metabolic signature and traditional prognostic factors was assessed using Cox proportional hazard models. Variables with *p* < 0.05 in the univariate analyses were used for further multivariate analysis. The final Cox model retained significant variables as independent predictors (*p* < 0.05), including the Met score (continuous variable), sex (male vs. female), overall stage (III vs. IVa), and EBV-DNA level (<4000 vs. ≥4000 copies/mL). For the multivariable Cox regression model, coefficients were used to construct the nomogram. The discrimination of the model predicting the 5-year PFS probability was evaluated using the C-index in the training and validation sets. Clinical usefulness was estimated by decision curve analysis (DCA). Finally, a nomogram was used to visualize the regression coefficients of the comprehensive model and was calibrated using a calibration plot [24].

### 2.8. Statistical Analysis

Data analyses were performed using the R software (version 4.1.0). Participants were randomly assigned to the training and validation sets in a ratio of 7:3 using the R package “IOBR” [25]. Metabolomic data were normalized using the Z-score method. Metabolites related to PFS were screened out by univariate Cox regression (FDR < 0.05), the *p*-value of the Cox proportional hazards regression model was obtained by the Wald test, and the R function p.adjust was used for the FDR by Benjamini–Hochberg method adjusted *p*-values. Based on the minimum Akaike information criteria (AIC), multivariate Cox regression analysis was performed to further narrow down the range of candidate metabolites related to PFS. The Kaplan–Meier analyses and log-rank tests were performed using the R package survival, and survival curves were visualized using the R package survminer. The time-dependent area under the ROC curve was computed using the R package “timeROC”. The C-index was calculated using the rcorr.cens function implemented in the Hmisc library. The bootstrap percentile method was used to calculate 95% confidence intervals. The rms package was used for nomograms and calibration curves. Kyoto Encyclopedia of Genes and Genomes (KEGG) pathway enrichment analysis was performed on the differential metabolites using the ClusterProfile package in R. All figures and heatmaps were produced using the ggplot2 and pheatmap packages in R, unless otherwise specified. All statistical tests were conducted with a two-sided significance level of 0.05.

## 3. Results

### 3.1. Patient Sets and Baseline Characteristics

For this study, we collected 320 pretreatment and non-distant metastatic LA-NPC serum samples. Table 1 shows the baseline characteristics of the patients in the training (*n* = 224) and test (*n* = 96) sets. There were no significant differences observed in baseline characteristics between the two sets in terms of age, sex, smoking, drinking, BMI, stage, EBV-DNA level, and some biomarkers that have been extensively investigated in NPC and are highly correlated with survival outcomes [34,35,36]. The median follow-up was 64.0 months (IQR = 33.2 to 68.4) in the training set and 65.7 months (IQR = 56.7 to 69.5) in the validation set.

### 3.2. Construction of the 9-Metabolite Signature

Widely targeted metabolite profiling of samples was performed using the self-built Metware database (MWDB) developed by MetWare Biotechnology Co. In total, 746 endogenous metabolites were detected using an UPLC–MS/MS system. Metabolite annotation details are described in Table S1.

**Figure 2:**
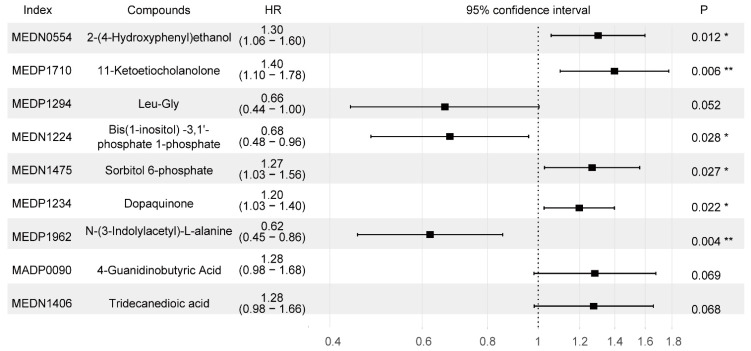
Forest plot of the nine selected metabolites related to the 5-year PFS. Index is the code of the metabolite in the Metware database. Compounds is the name of the substance corresponding to the index. HRs (95% CI) were calculated by applying a Cox regression analysis. Abbreviations: HR, hazard ratio. * indicates *p* < 0.05, ** indicates *p* < 0.01.

Twenty-seven different metabolites were significantly associated with PFS in the training set (FDR < 0.05) (Table S2). Using an Akaike information criterion (AIC)-based stepwise selection method, we computed an optimal predictive model including nine metabolites (Figure 2). Among these metabolites, 2-(4-Hydroxyphenyl) ethanol, 11-Ketoetiocholanolone, 2-(4-Hydroxyphenyl) ethanol, 11-Ketoetiocholanolone4-Guanidinobutyric acid, and tridecanedioic acid were negatively correlated with PFS, whereas Leu-Gly, bis (1-inositol) -3,1′-phosphate 1-phosphate, and N-(3-Indolylacetyl)-L-alanine were correlated with positive clinical outcomes.

We constructed a metabolic risk score (Met score) with the Cox regression coefficients of each metabolite in the 9-metabolite signature. Met score = (0.2630 × abundance of MEDN0554) + (0.3352 × abundance of MEP1710) − (0.4111 × abundance of MEDP1294) − (0.3887 × abundance of MEDN1224) + (0.2368 × abundance of MEDN1475) + (0.1804 × abundance of MEDP1234) − (0.4752 × abundance of MEDP1962) + (0.2491 × abundance of MADP0090) + (0.2443 × abundance of MEDN1406).

**Figure 3:**
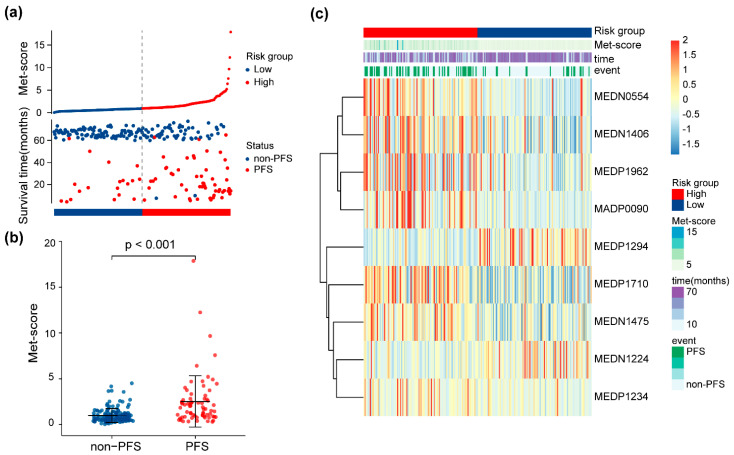
Risk score analysis of the 9-metabolite signature. (**a**) Met score for each patient in the training set. The dotted line represents the cutoff (0.92) used to divide patients into the high- and low-risk groups in the training set. (**b**) Distributions of Met score between patients with different PFS statuses. (**c**) Heatmap of expression levels of the nine PFS-related metabolites from the signature between low- and high-risk patients.

The Met score for each patient in the training set is shown in Figure 3a. Patients were stratified into high- and low-risk groups based on the median Met score (cutoff = 0.92). The Met-score distribution was significantly different between the groups with and without progression (*p* < 0.001, Figure 3b). A heatmap of significantly different metabolites is shown in Figure 3c to facilitate understanding of the prognostic signature.

### 3.3. Prognostic Validation of 9-Metabolite Signature

**Figure 4:**
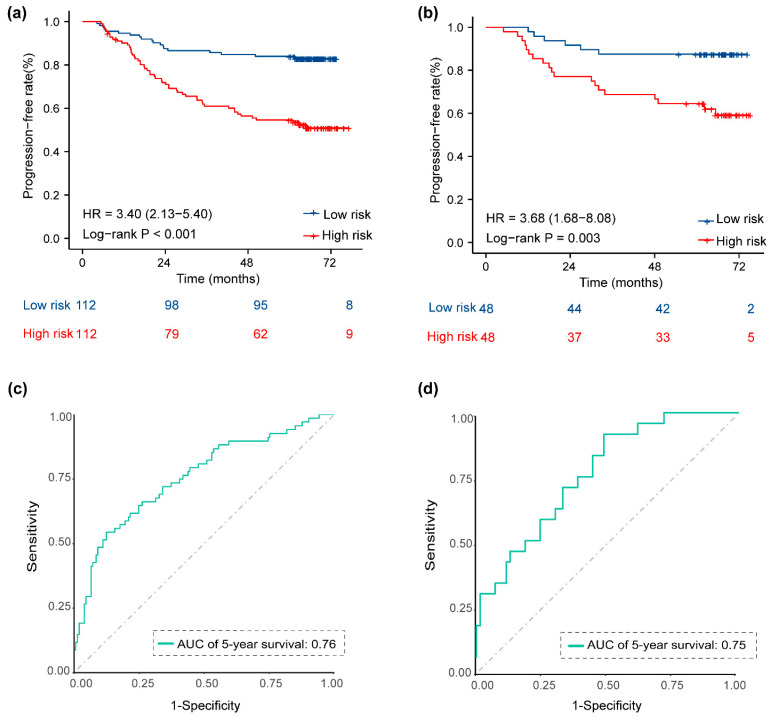
Kaplan–Meier survival curves and ROC curves for low- and high-risk groups based on the 9-metabolite signature to predict 5-year PFS. Kaplan–Meier curves for PFS stratified by the 9-metabolite signature in the training set (**a**) and the validation set (**b**). ROC curves comparing the predictive power of the 9-metabolite signature for PFS in the training set (**c**) and the validation set (**d**). Abbreviations: PFS, progression-free survival; ROC, receiver operating characteristic.

We assessed the potential relationship between the 9-metabolite signature and PFS in the training set and validated it in the validation set. KM survival analysis was used to compare survival differences between the two sets. Patients were divided into low- and high-risk groups based on the median Met score (cutoff = 0.92). The results of the KM curve showed that the prognosis of the high-risk group was worse than that of the low-risk groups in the training set (Figure 4a, log-rank *p* < 0.001; HR = 3.40, 95% CI = 2.13–5.40). Five-year PFS was 53.7% (95% CI 45.12–63.86) for the high-risk group and 83.0% (95% CI 76.31–90.26) for the low-risk group. When patients in the validation set (*n*= 96) were stratified according to their Met score with the same cutoff values used in the training set, the 5-year PFS rates for the low-risk (*n*= 52, 54.2%) and high-risk (*n*= 44, 45.8%) patient groups were 90.9% (95% CI 82.8–99.8) and 58.6% (95% CI 46.4–74.1), respectively (*p* = 0.003, log-rank test, HR = 3.68, 95% CI = 1.68–8.08 (Figure 4b)). Time-dependent ROC curves for each specified time point were used to assess the accuracy of the 9-metabolite signature in predicting PFS in LA-NPC patients. The area under the curve (AUC), calculated for predicting the 5-year PFS in the training set and validation set, was 0.76 (Figure 4c) and 0.75 (Figure 4d), respectively. Furthermore, the C-index of this model, based on metabolites, also validated that the model had good prognostic power (Table 2. Next, we explored the prognostic value of the 9-metabolite signature on other endpoints. In the training set, a high Met score was associated with a higher risk of distant metastasis, local recurrence, and death (HR 2.76, 95% CI 1.52–5.03, *p* = 0.002, Supplemental Figure S3a; HR 4.21, 95% CI 2.12–8.33, *p* < 0.001, Figure S3c; HR 2.45, 95% CI 1.26–4.75, *p* = 0.012, Figure S3e). This trend was also consistently observed for DMFS, LRFS, and OS in the validation set, although it was not statistically significant (Figure S3b,d,e). The ROC curve shown in Figure S4 indicates moderate to good performance in predicting 5-year DMFS (AUC= 0.73; AUC = 0.68), 5-year LRFS (AUC = 0.75; AUC = 0.75), and 5-year OS (AUC = 0.75; AUC = 0.58) in the training and validation sets. These results demonstrate the robustness and accuracy of the 9-metabolite signature for predicting patient prognosis.

### 3.4. Development of an Individualized Prognostic Model

We developed a prognostic model considering both the 9-metabolite signature and other clinical factors for predicting PFS. In univariate analysis, Met score and clinical factors including EBV-DNA level, overall stage, smoking, sex, and CRP were found to be significantly associated with the 5-year PFS (Table 3; all *p* < 0.05). Variables univariately correlated with PFS were entered into a multivariate Cox proportional hazards model. The Met score (HR, 1.18; 95% CI, 1.11–1.26; *p* < 0.001), overall stage (HR, 1.93; 95% CI, 1.19–3.15; *p* = 0.008), sex (HR, 2.44; 95% CI, 1.25–4.80; *p* < 0.001), and EBV-DNA (HR, 2.33; 95% CI, 1.44–3.79; *p* < 0.001) were still independent predictors of PFS after adjusting for other various cofactors (Table 3). The results showed that our constructed 9-metabolite signature could serve as a robust and novel biomarker for predicting prognosis.

### 3.5. Performance and Validation of the Prognostic Nomogram

**Figure 5:**
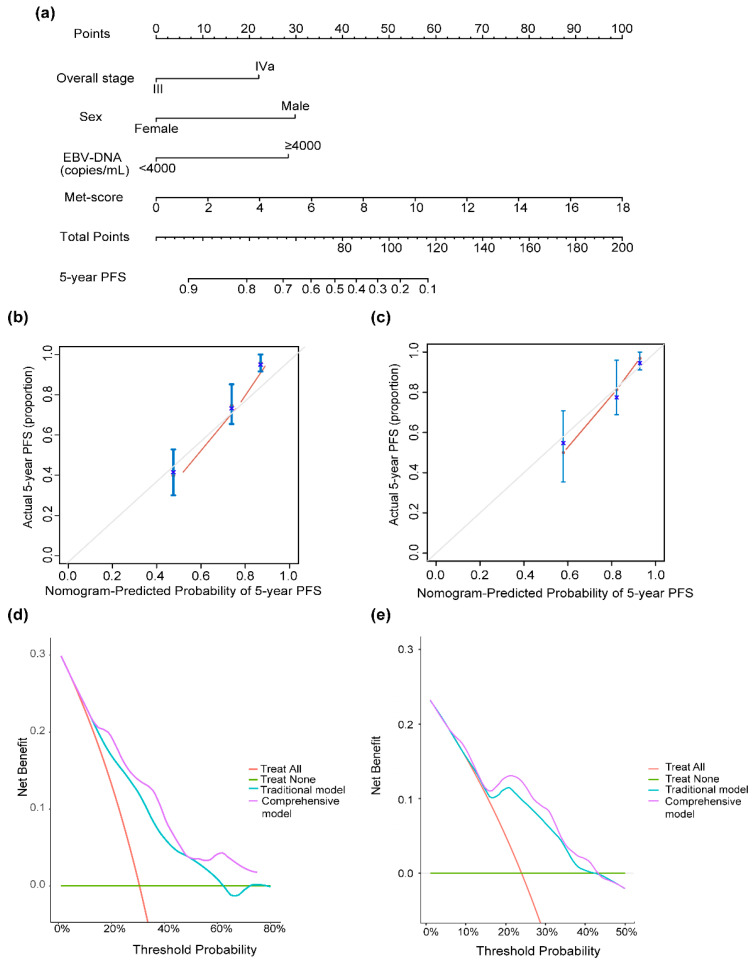
(**a**) Nomogram for 5-year PFS in patients with locally advanced nasopharyngeal carcinoma. Calibration curve of nomogram models for predicting 5-year PFS in the training set (**b**) and the validation set (**c**). Decision curve analysis of the nomogram for the PFS in the training set (**d**) and the validation set (**e**). The nomogram incorporating sex, overall stage, EBV DNA, and Met score was developed and presented. Abbreviations: EBV DNA, Epstein–Barr virus DNA.

A nomogram for individualized PFS prediction was constructed using these variables (Figure 5a). Compared with the traditional prognostic model—including sex, overall stage, and pretreatment EBV-DNA—the additional combination of the Met score showed better discrimination in the training (C-index, 0.77 vs. 0.71) and validation sets (C-index, 0.72 vs. 0.67) (Table 2). The calibration curves plotted at the 5-year time points visually confirmed a good fit between the predicted nomogram-predicted PFS probability and observed PFS rates in the two sets (Figure 5b,c). DCA was used to evaluate the clinical utility of the nomogram. The DCA for the 5-year PFS nomogram showed that for a threshold probability >15%, using the comprehensive nomogram model to predict the 5-year PFS could add more benefits than the traditional model in the training set (Figure 5d), which are then identified in the validation set (Figure 5e).

### 3.6. Metabolite Set Enrichment Analysis

**Figure 6:**
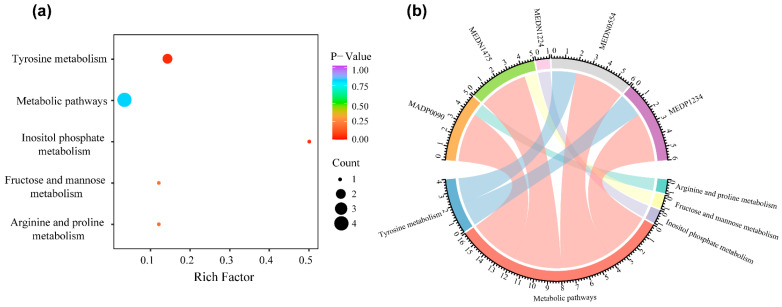
KEGG pathway enrichment of metabolites in the 9-metabolite signature. (**a**) Bubble plot of KEGG pathway enrichment analyses. (**b**) Chord diagram for KEGG pathway. Abbreviations: KEGG, Kyoto Encyclopedia of Genes and Genomes.

The metabolites that were significantly related to PFS were analyzed for enrichment in KEGG pathways using the KEGG compound database. We observed that metabolites were enriched in four pathways: tyrosine metabolism, inositol phosphate metabolism, fructose and mannose metabolism, and arginine and proline metabolism (Figure 6b). The identified PFS-related metabolites were widely involved in amino acid and energy metabolism. The tyrosine metabolism pathway was the most significantly enriched pathway (*p* < 0.05, Figure 6a).

## 4. Discussion

In our study, we developed an individualized biomarker named the 9-metabolite signature for predicting the PFS of patients with LA-NPC based on metabolomics. A prognostic model integrating metabolites and important clinical characteristics was proposed with better performance in the training and validation sets, compared with the model based on conventional clinical data. The study focused on investigating new prognostic factors to complement the TNM staging system and improve the accurate prognosis of patients with LA-NPC for providing aggressive treatment plans for high-risk groups. To our knowledge, this is the first large-scale study to focus exclusively on the relationship between patient prognosis and pretreatment serum metabolomics in advanced NPC.

The reprogramming of cellular metabolism is one of the hallmarks of cancer transformation and progression [23,37]. The metabolites are closely related to the phenotype and dynamic changes in the organism, which are generally used as biomarkers for cancer diagnosis, progression, and assessment of therapeutic efficacy [22,38]. Thus, high-throughput metabolomics can provide more comprehensive information about tumors. Many studies have explored the association between metabolic characteristics and the occurrence and progression of various types of cancer [27,39].

However, the metabolomics for NPC is still in its infancy. Previous studies mainly focused on the early diagnosis of NPC, which detected serum metabolites in a crowd of small-scale patients with NPC and normal controls by GC-MS-based metabolic profiling [29,30]. Research regarding the prognostic significance of metabolomics in the field of NPC is less extensive. Tang et al. [29] observed changes of metabolites in 19 patients with primary NPC at three time periods after radiotherapy. They found the high expression of kynurenine, Nacetylglucosamine, N-acetylglucosaminylamine, and hydroxyphenylpyruvate was related to cancer recurrence and distant metastasis. The drawbacks of these works include the use of a small cohort and the lack of an independent validation set, the relatively short follow-up period, and the absence of evaluation of other clinical variables related to survival. Compared with earlier studies, our study has several differences and advantages. We analyzed a relatively larger cohort, with hundreds of samples available for metabolomic analysis, and performed a 5-year follow-up. In addition, a widely targeted metabolomics approach using ultra performance liquid chromatography—tandem mass spectrometry (UPLC–MS/MS) was used to investigate serum metabolites and produce a metabolic profile in LA-NPC patients in our study. This technology not only enables high-throughput analysis but also ensures reliable high-quality data with higher resolution separations [40]. It broadens the scope of potential biomarker discovery by improving the detection limit of low-abundance metabolites. Additionally, we added the value of the Met score to clinical data in developing the prognostic model and assessed its performance in a validation set. Notably, the Met score remained an independent prognostic factor after the inclusion of conventional clinical biomarkers as covariates in the multivariable analysis. In our study, we combined a newly developed metabolomics-based biomarker with reliable clinical prognostic factors including sex, overall stage, and pretreatment EBV-DNA level to add more value for prognostic prediction and clinical application [41,42,43]. This complementary ability seems to be practical because serum metabolomic profiling is more convenient and cheaper than other omics methods. This nomogram provides a simple and accurate tool for predicting prognosis in patients with LA-NPC before treatment.

Most of the metabolites screened in this study have been reported to be related to the occurrence and development of diseases in previous studies, which is consistent with our results. Luo et al. found that 2-(4-Hydroxyphenyl)ethanol was involved in inflammation and NF-κB activation [44]. Etiocholanolone is an excreted metabolite of testicular hormone and 11-Ketoetiocholanolone is a metabolite of Etiocholanolone [45]. We observed that 11-Ketoetiocholanolone has a significant negative correlation with a good prognosis in LA-NPC, which indicates that males always have a poorer prognosis. Zeebroeck discovered that the dipeptide L-Leu-Gly, a non-transport agonist of the active transporter receptor, could activate the PKA pathway, which may promote the proliferation of tumor cells [46]. Studies have demonstrated that dopaquinone is an intermediate in melanogenesis that can produce toxic oxygen free radicals and cause cellular damage [47]. In addition, 4-Guanidinobutanoic acid, a gamma-amino acid and uremia toxin, has been shown to increase in early renal cell carcinoma compared to that in healthy humans [48]. Tridecanedioic acid, an unusual fatty acid related to peroxisomal disorders, can discriminate healthy people from patients with psoriatic arthritis, as reported in previous literature [49,50]. Regrettably, except for those mentioned above, there are few relevant literature reports on the relationship between bis (1-inositol)-3,1′-phosphate 1-phosphate, sorbitol 6-phosphate, and N-(3-Indolylacetyl)-L-alanine and diseases. Although the roles of these metabolites in NPC or other diseases are currently unclear, our results suggest that further research is needed.

KEGG enrichment analysis identified metabolites annotated using the KEGG compound database. The tyrosine metabolism pathway was the most significantly enriched pathway (*p* < 0.05). Several studies have confirmed that tyrosine metabolism is an important process that is often dysregulated in cancer development and progression [51,52,53]. Cheng et al. [54] observed that tyrosine metabolism is disturbed in esophageal squamous cell carcinoma patients, and the metabolites involved in the tyrosine pathway can be used as diagnostic biomarkers of the disease. These results suggest a relationship between tyrosine metabolism and nasopharyngeal carcinoma that needs to be investigated in future studies.

Finally, we developed an integrated prognostic model incorporating 9-metabolite signature, gender, overall stage, and pretreatment EBV-DNA levels. In addition to the Met score, other traditional prognostic factors included in this integrated prognostic model have been proven to be powerful independent prognostic factors for NPC in previous studies. Previous research has shown that male patients are more likely to exhibit inferior overall survival and disease progression-free survival rates compared to female patients [41,55], which is consistent with our findings. Male and female patients with NPC may exhibit differences in tumor behavior, indicating potential biological distinctions. These gender disparities in NPC incidence and prognosis may be influenced by genetic variations influenced by hormonal factors. The TNM stage remains an important clinical factor that affects cancer patient prognosis, as it dictates the overall clinical stage [42]. Furthermore, multiple studies have shown that pre-treatment EBV-DNA is closely correlated with the tumor burden and prognosis in patients with NPC [56,57]. In our study, we combined these reliable clinical prognostic factors with a newly developed metabolomics-based biomarker to enhance the prognostic prediction and clinical application of our model.

The results showed that the integrated model was better than the traditional model in predicting 5-year PFS in locally advanced nasopharyngeal carcinoma (training set: C-index 0.77 vs. 0.71; validation set: C-index 0.72 vs. 0.67). The main reason for this is the limitations of the TNM staging system [42,43]. This staging system mainly focuses on the macroscopic information of tumors provided by imaging but omits the biological characteristics of tumors and cannot reflect tumor heterogeneity. As a powerful tool, metabolomics can provide more comprehensive tumor information through the analysis of circulating metabolites in vivo. Therefore, it can complement traditional prognostic factors, improve the accuracy of prognosis prediction, and provide active treatment options for high-risk groups in a timely manner. In addition, the metabolic signature developed in this study has certain clinical application value. Compared with other omics, serum metabolomics is convenient to obtain materials, and the price is lower, which will not increase the economic burden of patients. This metabolic signature may provide a simple and accurate prognostic prediction tool for locally advanced NPC patients before treatment.

The present study had certain limitations. First, all study participants were enrolled from a single institution in endemic NPC areas, which may have enhanced the standardized sample collection. However, these observations require confirmation in an external validation set so that our prognostic model could become more widely applicable. Second, the biological functions and underlying mechanisms of these significant metabolites related to poor prognosis require further investigation. Third, as the metabolome is highly dynamic and changes in response to the occurrence and progression of the disease, longitudinal, continuous, and prospective metabolomic profiling during the treatment process is necessary to capture more precise biological information. Finally, we will use multi-omics data to explore new insights into survival prediction in NPC in the future.

## 5. Conclusions

In conclusion, we developed and validated a signature based on a panel of metabolites as a novel biomarker to predict PFS in patients with LA-NPC (stages III–IVa). Nine metabolites that were closely associated with PFS were identified via the application of highly sensitive UPLC–MS/MS-based widely targeted metabolomic analysis. The 9-metabolite signature can effectively distinguish populations at high or low risk for LA-NPC so that appropriate treatment strategies can be implemented. The prognostic model combining the metabolic signature with the staging system and clinical data showed robust predictive power for pretreatment PFS, which may have translational implications in clinical practice.

## Figures and Tables

**Table 1:** Baseline information of the training and internal validation sets.

Characteristics	Training Set (*n* = 224)	Test Set (*n* = 96)	*p*-Values
	No. (%)	No. (%)	
Age (years)			0.329
Age < 45	113 (50.4%)	42 (43.8%)	
Age ≥ 45	111 (49.6%)	54 (56.2%)	
Sex			0.806
Female	63 (28.1%)	25 (26%)	
Male	161 (71.9%)	71 (74%)	
T stage			0.495
T1	18 (8%)	6 (6.2%)	
T2	21 (9.4%)	13 (13.5%)	
T3	127 (56.7%)	48 (50%)	
T4	58 (25.9%)	29 (30.2%)	
N stage			0.128
N0	16 (7.1%)	14 (14.6%)	
N1	94 (42%)	36 (37.5%)	
N2	73 (32.6%)	25 (26%)	
N3	41 (18.3%)	21 (21.9%)	
Overall stage			0.902
III	127 (56.7%)	53 (55.2%)	
IVA	97 (43.3%)	43 (44.8%)	
Smoking			0.745
Yes	139 (62.1%)	57 (59.4%)	
No	85 (37.9%)	39 (40.6%)	
Drinking			0.444
Yes	187 (83.5%)	76 (79.2%)	
No	37 (16.5%)	20 (20.8%)	
Family history of NPC			0.012
Yes	12 (5.4%)	14 (14.6%)	
No	212 (94.6%)	82 (85.4%)	
BMI (kg/m^2^)			0.85
<18.5	17 (7.6%)	6 (6.2%)	
≥18.5	207 (92.4%)	90 (93.8%)	
EBV DNA (copies/mL)			0.428
<4000	142 (63.4%)	66 (68.8%)	
≥4000	82 (36.6%)	30 (31.2%)	
HGB (g/L)			0.508
<120	18 (8%)	5 (5.2%)	
≥120	206 (92%)	91 (94.8%)	
ALB (g/L)			0.98
<43.3	86 (38.4%)	36 (37.5%)	
≥43.3	138 (61.6%)	60 (62.5%)	
LDH (U/L)			0.263
<245	215 (96%)	89 (92.7%)	
≥245	9 (4%)	7 (7.3%)	
CRP (mg/L)			0.104
<3	160 (71.4%)	59 (61.5%)	
≥3	64 (28.6%)	37 (38.5%)	

Abbreviations: NPC, nasopharyngeal carcinoma; BMI, body mass index; EBV DNA, Epstein–Barr virus DNA; HGB, hemoglobin; ALB, albumin; LDH, lactate dehydrogenase; CRP, C-reactive protein.

**Table 2:** C-index and corresponding 95% CI of all prognostic models.

Models	Training Set	Validation Set
	C-Index (95% CI)	C-Index (95% CI)
Traditional model	0.71 (0.66–0.77)	0.67 (0.56–0.78)
Metabolites model	0.71 (0.64–0.77)	0.73 (0.63–0.82)
Comprehensive model	0.77 (0.72–0.81)	0.72 (0.62–0.81)

Abbreviations: C-index, concordance index; CI, confidence interval.

**Table 3:** Results of univariate and stepwise multivariate analyses in the training set.

Characteristics	Univariate Model		Multivariate Model	
	HR (95% CI)	*p*	HR (95% CI)	*p*
Age (years)				
Age < 45	Reference			
Age ≥ 45	1.01 (0.63–1.57)	0.98	NA	NA
Sex				0.009
Female	Reference		Reference	
Male	2.86 (1.47–5.59)	0.002	2.44 (1.25–4.80)	
Overall stage				0.008
III	Reference		Reference	
IVA	2.42 (1.51–3.89)	<0.001	1.93 (1.19–3.15)	
Smoking				
Yes	Reference			
No	2.28 (1.43–3.62)	0.001	NA	NA
Drinking				
Yes	Reference			
No	1.49 (0.82–2.56)	0.202	NA	NA
Family history of cancer				
Yes	Reference			
No	0.88 (0.49–1.57)	0.66	NA	NA
BMI (kg/m^2^)				
<18.5	Reference			
≥18.5	0.73 (0.34–1.60)	0.439	NA	NA
EBV-DNA (copies/mL)				<0.001
<4000	Reference		Reference	
≥4000	1.81 (1.37–2.38)	<0.001	2.34 (1.44–3.79)	
HGB (g/L)				
<120	Reference			
≥120	1.22 (0.49–3.02)	0.671	NA	NA
ALB (g/L)				
<43.3	Reference			
≥43.3	1.19 (0.73–1.94)	0.474	NA	NA
LDH (U/L)				
<245	Reference			
≥245	1.41 (0.44–4.47)	0.563	NA	NA
CRP (mg/L)				
<3	Reference			
≥3	1.93 (1.21–3.09)	0.006	NA	NA
Met score	1.26 (1.18–1.34)	<0.001	1.18 (1.11–1.26)	<0.001

Abbreviations: BMI, body mass index; EBV-DNA, Epstein–Barr virus DNA; HGB, hemoglobin; ALB, albumin; LDH, lactate dehydrogenase; CRP, C-reactive protein; "NA" represents variables that were not included in the final multivariable model.

## Data Availability

The data underlying this article will be shared on reasonable request to the corresponding author.

## Supplementary Materials

The following supporting information can be downloaded at: https://www.mdpi.com/article/10.3390/life13051167/s1, Supplementary Methods: Details of the widely targeted metabolomic profiling analysis and quality control analysis. Figure S1: Heatmap visualization of 746 identified metabolites; Figure S2: Sample quality control analysis; Figure S3: Kaplan–Meier curves of the 9-metabolite signature for predicting secondary endpoints in training and validation sets; Figure S4: Receiver operating characteristic curve of the 9-metabolite signature for predicting secondary endpoints in training and validation sets. Table S1: Specific information of 746 metabolites; Table S2: Information of 27 metabolites associated with PFS by univariate Cox regression.

## References

[1] Chen Y.-P., Chan A.T.C., Le Q.-T., Blanchard P., Sun Y., Ma J. (2019). Nasopharyngeal Carcinoma. Lancet.

[2] Pua K.C., Khoo A.S.B., Yap Y.Y., Subramaniam S.K., Ong C.A., Gopala Krishnan G., Shahid H. (2008). Malaysian Nasopharyngeal Carcinoma Study Group Nasopharyngeal Carcinoma Database. Med. J. Malaysia.

[3] Tang L.-L., Chen Y.-P., Mao Y.-P., Wang Z.-X., Guo R., Chen L., Tian L., Lin A.-H., Li L., Sun Y. (2017). Validation of the 8th Edition of the UICC/AJCC Staging System for Nasopharyngeal Carcinoma From Endemic Areas in the Intensity-Modulated Radiotherapy Era. J. Natl. Compr. Cancer Netw..

[4] Chen Y.-P., Liu X., Zhou Q., Yang K.-Y., Jin F., Zhu X.-D., Shi M., Hu G.-Q., Hu W.-H., Sun Y. (2021). Metronomic Capecitabine as Adjuvant Therapy in Locoregionally Advanced Nasopharyngeal Carcinoma: A Multicentre, Open-Label, Parallel-Group, Randomised, Controlled, Phase 3 Trial. Lancet.

[5] Caudell J.J., Gillison M.L., Maghami E., Spencer S., Pfister D.G., Adkins D., Birkeland A.C., Brizel D.M., Busse P.M., Cmelak A.J. (2022). NCCN Guidelines^®^ Insights: Head and Neck Cancers, Version 1.2022. J. Natl. Compr. Cancer Netw..

[6] Wang H.-Y., Sun B.-Y., Zhu Z.-H., Chang E.T., To K.-F., Hwang J.S.G., Jiang H., Kam M.K.-M., Chen G., Cheah S.-L. (2011). Eight-Signature Classifier for Prediction of Nasopharyngeal [Corrected] Carcinoma Survival. J. Clin. Oncol..

[7] Hielscher A., Gerecht S. (2015). Hypoxia and Free Radicals: Role in Tumor Progression and the Use of Engineering-Based Platforms to Address These Relationships. Free Radic. Biol. Med..

[8] Ma T., Zhang P., Hou Y., Ning H., Wang Z., Huang J., Gao M. (2018). “Smart” Nanoprobes for Visualization of Tumor Microenvironments. Adv. Healthc. Mater..

[9] Kreso A., Dick J.E. (2014). Evolution of the Cancer Stem Cell Model. Cell Stem Cell.

[10] Brown J.M. (2002). Tumor Microenvironment and the Response to Anticancer Therapy. Cancer Biol. Ther..

[11] Mimmi S., Lombardo N., Maisano D., Piazzetta G., Pelaia C., Pelaia G., Greco M., Foti D., Dattilo V., Iaccino E. (2022). Spotlight on a Short-Time Treatment with the IL-4/IL-13 Receptor Blocker in Patients with CRSwNP: MicroRNAs Modulations and Preliminary Clinical Evidence. Genes.

[12] Kim K.Y., Le Q.-T., Yom S.S., Pinsky B.A., Bratman S.V., Ng R.H.W., El Mubarak H.S., Chan K.C.A., Sander M., Conley B.A. (2017). Current State of PCR-Based Epstein-Barr Virus DNA Testing for Nasopharyngeal Cancer. J. Natl. Cancer Inst..

[13] Lv S.-H., Li W.-Z., Liang H., Liu G.-Y., Xia W.-X., Xiang Y.-Q. (2021). Prognostic and Predictive Value of Circulating Inflammation Signature in Non-Metastatic Nasopharyngeal Carcinoma: Potential Role for Individualized Induction Chemotherapy. J. Inflamm. Res..

[14] Diakos C.I., Charles K.A., McMillan D.C., Clarke S.J. (2014). Cancer-Related Inflammation and Treatment Effectiveness. Lancet. Oncol..

[15] Zhang B., Tian J., Dong D., Gu D., Dong Y., Zhang L., Lian Z., Liu J., Luo X., Pei S. (2017). Radiomics Features of Multiparametric MRI as Novel Prognostic Factors in Advanced Nasopharyngeal Carcinoma. Clin. Cancer Res..

[16] Qiang M., Li C., Sun Y., Sun Y., Ke L., Xie C., Zhang T., Zou Y., Qiu W., Gao M. (2021). A Prognostic Predictive System Based on Deep Learning for Locoregionally Advanced Nasopharyngeal Carcinoma. J. Natl. Cancer Inst..

[17] Peng H., Dong D., Fang M.-J., Li L., Tang L.-L., Chen L., Li W.-F., Mao Y.-P., Fan W., Liu L.-Z. (2019). Prognostic Value of Deep Learning PET/CT-Based Radiomics: Potential Role for Future Individual Induction Chemotherapy in Advanced Nasopharyngeal Carcinoma. Clin. Cancer Res..

[18] Le Q.-T., Jones C.D., Yau T.-K., Shirazi H.A., Wong P.H., Thomas E.N., Patterson B.K., Lee A.W.M., Zehnder J.L. (2005). A Comparison Study of Different PCR Assays in Measuring Circulating Plasma Epstein-Barr Virus DNA Levels in Patients with Nasopharyngeal Carcinoma. Clin. Cancer Res..

[19] Parmar C., Leijenaar R.T.H., Grossmann P., Velazquez E.R., Bussink J., Rietveld D., Rietbergen M.M., Haibe-Kains B., Lambin P., Aerts H.J. (2015). Radiomic Feature Clusters and Prognostic Signatures Specific for Lung and Head & Neck Cancer. Sci. Rep..

[20] Huang Y.-Q., Liang C.-H., He L., Tian J., Liang C.-S., Chen X., Ma Z.-L., Liu Z.-Y. (2016). Development and Validation of a Radiomics Nomogram for Preoperative Prediction of Lymph Node Metastasis in Colorectal Cancer. J. Clin. Oncol. Off. J. Am. Soc. Clin. Oncol..

[21] Leung S.F., Chan K.C.A., Ma B.B., Hui E.P., Mo F., Chow K.C.K., Leung L., Chu K.W., Zee B., Lo Y.M.D. (2014). Plasma Epstein-Barr Viral DNA Load at Midpoint of Radiotherapy Course Predicts Outcome in Advanced-Stage Nasopharyngeal Carcinoma. Ann. Oncol. Off. J. Eur. Soc. Med. Oncol..

[22] Ussher J.R., Elmariah S., Gerszten R.E., Dyck J.R.B. (2016). The Emerging Role of Metabolomics in the Diagnosis and Prognosis of Cardiovascular Disease. J. Am. Coll. Cardiol.

[23] Pavlova N.N., Zhu J., Thompson C.B. (2022). The Hallmarks of Cancer Metabolism: Still Emerging. Cell Metab..

[24] Li X., Wenes M., Romero P., Huang S.C.-C., Fendt S.-M., Ho P.-C. (2019). Navigating Metabolic Pathways to Enhance Antitumour Immunity and Immunotherapy. Nat. Rev. Clin. Oncol..

[25] DeBerardinis R.J., Chandel N.S. (2016). Fundamentals of Cancer Metabolism. Sci. Adv..

[26] Schmidt D.R., Patel R., Kirsch D.G., Lewis C.A., Vander Heiden M.G., Locasale J.W. (2021). Metabolomics in Cancer Research and Emerging Applications in Clinical Oncology. CA Cancer J. Clin..

[27] Huang S., Guo Y., Li Z.-W., Shui G., Tian H., Li B.-W., Kadeerhan G., Li Z.-X., Li X., Zhang Y. (2021). Identification and Validation of Plasma Metabolomic Signatures in Precancerous Gastric Lesions That Progress to Cancer. JAMA Netw. Open.

[28] Bachmayr-Heyda A., Aust S., Auer K., Meier S.M., Schmetterer K.G., Dekan S., Gerner C., Pils D. (2017). Integrative Systemic and Local Metabolomics with Impact on Survival in High-Grade Serous Ovarian Cancer. Clin. Cancer Res..

[29] Tang F., Xie C., Huang D., Wu Y., Zeng M., Yi L., Wang Y., Mei W., Cao Y., Sun L. (2011). Novel Potential Markers of Nasopharyngeal Carcinoma for Diagnosis and Therapy. Clin. Biochem..

[30] Yi L., Song C., Hu Z., Yang L., Xiao L., Yi B., Jiang W., Cao Y., Sun L. (2014). A Metabolic Discrimination Model for Nasopharyngeal Carcinoma and Its Potential Role in the Therapeutic Evaluation of Radiotherapy. Metabolomics.

[31] Liao Z., Zhao L., Zhong F., Zhou Y., Lu T., Liu L., Gong X., Li J., Rao J. (2023). Serum and Urine Metabolomics Analyses Reveal Metabolic Pathways and Biomarkers in Relation to Nasopharyngeal Carcinoma. Rapid Commun. Mass Spectrom..

[32] Chen W., Gong L., Guo Z., Wang W., Zhang H., Liu X., Yu S., Xiong L., Luo J. (2013). A Novel Integrated Method for Large-Scale Detection, Identification, and Quantification of Widely Targeted Metabolites: Application in the Study of Rice Metabolomics. Mol. Plant.

[33] Xu J., Li J., Zhang R., He J., Chen Y., Bi N., Song Y., Wang L., Zhan Q., Abliz Z. (2019). Development of a Metabolic Pathway-Based Pseudo-Targeted Metabolomics Method Using Liquid Chromatography Coupled with Mass Spectrometry. Talanta.

[34] Tang L.-Q., Li C.-F., Li J., Chen W.-H., Chen Q.-Y., Yuan L.-X., Lai X.-P., He Y., Xu Y.-X.-X., Hu D.-P. (2016). Establishment and Validation of Prognostic Nomograms for Endemic Nasopharyngeal Carcinoma. J. Natl. Cancer Inst..

[35] Li J., Chen S., Peng S., Liu Y., Xing S., He X., Chen H. (2018). Prognostic Nomogram for Patients with Nasopharyngeal Carcinoma Incorporating Hematological Biomarkers and Clinical Characteristics. Int. J. Biol. Sci.

[36] Zhang L.-L., Xu F., Song D., Huang M.-Y., Huang Y.-S., Deng Q.-L., Li Y.-Y., Shao J.-Y. (2020). Development of a Nomogram Model for Treatment of Nonmetastatic Nasopharyngeal Carcinoma. JAMA Netw. Open.

[37] Vander Heiden M.G., DeBerardinis R.J. (2017). Understanding the Intersections between Metabolism and Cancer Biology. Cell.

[38] Weiner J., Maertzdorf J., Sutherland J.S., Duffy F.J., Thompson E., Suliman S., McEwen G., Thiel B., Parida S.K., Zyla J. (2018). Metabolite Changes in Blood Predict the Onset of Tuberculosis. Nat. Commun..

[39] Farshidfar F., Weljie A.M., Kopciuk K.A., Hilsden R., McGregor S.E., Buie W.D., MacLean A., Vogel H.J., Bathe O.F. (2016). A Validated Metabolomic Signature for Colorectal Cancer: Exploration of the Clinical Value of Metabolomics. Br. J. Cancer.

[40] Yin P., Xu G. (2013). Metabolomics for Tumor Marker Discovery and Identification Based on Chromatography-Mass Spectrometry. Expert Rev. Mol. Diagn..

[41] Li W.-Z., Lv S.-H., Liu G.-Y., Liang H., Xia W.-X., Xiang Y.-Q. (2021). Age-Dependent Changes of Gender Disparities in Nasopharyngeal Carcinoma Survival. Biol. Sex. Differ..

[42] Bossi P., Chan A.T., Licitra L., Trama A., Orlandi E., Hui E.P., Halámková J., Mattheis S., Baujat B., Hardillo J. (2021). Nasopharyngeal Carcinoma: ESMO-EURACAN Clinical Practice Guidelines for Diagnosis, Treatment and Follow-Up†. Ann. Oncol..

[43] Kim K.Y., Le Q.-T., Yom S.S., Ng R.H.W., Chan K.C.A., Bratman S.V., Welch J.J., Divi R.L., Petryshyn R.A., Conley B.A. (2017). Clinical Utility of Epstein-Barr Virus DNA Testing in the Treatment of Nasopharyngeal Carcinoma Patients. Int. J. Radiat. Oncol. Biol. Phys..

[44] Luo G., Huang Y., Mo D., Ma N., Gao F., Song L., Sun X., Xu X., Liu L., Huo X. (2018). Tyrosol Attenuates Pro-Inflammatory Cytokines from Cultured Astrocytes and NF-ΚB Activation in in Vitro Oxygen Glucose Deprivation. Neurochem. Int..

[45] Li P., Bracamontes J., Katona B.W., Covey D.F., Steinbach J.H., Akk G. (2007). Natural and Enantiomeric Etiocholanolone Interact with Distinct Sites on the Rat Alpha1beta2gamma2L GABAA Receptor. Mol. Pharmacol..

[46] Xiang S., Dauchy R.T., Hauch A., Mao L., Yuan L., Wren M.A., Belancio V.P., Mondal D., Frasch T., Blask D.E. (2015). Doxorubicin Resistance in Breast Cancer Is Driven by Light at Night-Induced Disruption of the Circadian Melatonin Signal. J. Pineal. Res..

[47] Onojafe I.F., Adams D.R., Simeonov D.R., Zhang J., Chan C.-C., Bernardini I.M., Sergeev Y.V., Dolinska M.B., Alur R.P., Brilliant M.H. (2011). Nitisinone Improves Eye and Skin Pigmentation Defects in a Mouse Model of Oculocutaneous Albinism. J. Clin. Investig..

[48] Bifarin O.O., Gaul D.A., Sah S., Arnold R.S., Ogan K., Master V.A., Roberts D.L., Bergquist S.H., Petros J.A., Edison A.S. (2021). Urine-Based Metabolomics and Machine Learning Reveals Metabolites Associated with Renal Cell Carcinoma Stage. Cancers.

[49] Looby N., Roszkowska A., Reyes-Garcés N., Yu M., Bączek T., Kulasingam V., Pawliszyn J., Chandran V. (2021). Serum Metabolic Fingerprinting of Psoriasis and Psoriatic Arthritis Patients Using Solid-Phase Microextraction-Liquid Chromatography-High-Resolution Mass Spectrometry. Metabolomics.

[50] Korman S.H., Mandel H., Gutman A. (2000). Characteristic Urine Organic Acid Profile in Peroxisomal Biogenesis Disorders. J. Inherit. Metab. Dis..

[51] Liu J., Mei J., Li S., Wu Z., Zhang Y. (2020). Establishment of a Novel Cell Cycle-Related Prognostic Signature Predicting Prognosis in Patients with Endometrial Cancer. Cancer Cell Int..

[52] Rodríguez-Hernández M.A., de la Cruz-Ojeda P., López-Grueso M.J., Navarro-Villarán E., Requejo-Aguilar R., Castejón-Vega B., Negrete M., Gallego P., Vega-Ochoa Á., Victor V.M. (2020). Integrated Molecular Signaling Involving Mitochondrial Dysfunction and Alteration of Cell Metabolism Induced by Tyrosine Kinase Inhibitors in Cancer. Redox Biol..

[53] Jin N., Bi A., Lan X., Xu J., Wang X., Liu Y., Wang T., Tang S., Zeng H., Chen Z. (2019). Identification of Metabolic Vulnerabilities of Receptor Tyrosine Kinases-Driven Cancer. Nat. Commun..

[54] Cheng J., Zheng G., Jin H., Gao X. (2017). Towards Tyrosine Metabolism in Esophageal Squamous Cell Carcinoma. Comb. Chem. High Throughput Screen.

[55] Xiao G., Cao Y., Qiu X., Wang W., Wang Y. (2013). Influence of Gender and Age on the Survival of Patients with Nasopharyngeal Carcinoma. BMC Cancer.

[56] Wang W.-Y., Twu C.-W., Chen H.-H., Jiang R.-S., Wu C.-T., Liang K.-L., Shih Y.-T., Chen C.-C., Lin P.-J., Liu Y.-C. (2013). Long-Term Survival Analysis of Nasopharyngeal Carcinoma by Plasma Epstein-Barr Virus DNA Levels. Cancer.

[57] Chen F.-P., Lin L., Liang J.-H., Tan S.H., Ong E.H.W., Luo Y.-S., Huang L., Sim A.Y.L., Wang H.-T., Gao T.-S. (2021). Development of a Risk Classification System Combining TN-Categories and Circulating EBV DNA for Non-Metastatic NPC in 10,149 Endemic Cases. Ther. Adv. Med. Oncol..

