# Supplementary material for: An Individualized Prognostic Model in Patients with Locoregionally Advanced Nasopharyngeal Carcinoma Based on Serum Metabolomic Profiling
Source: Life (Basel). 2023 May 11;13(5):1167. doi: 10.3390/life13051167 (PMC10224521; doi:10.3390/life13051167)
Supplement: Supplementary file 1 [file life-13-01167-s001.zip › life-2333724-supplementary.pdf]

Supplemental methods

Details of the widely targeted metabolomic profiling analysis

The chromatographic and MS conditions were as follows:

(1) T3 UPLC Conditions: UPLC: column, Waters ACQUITY UPLC HSS T3 C18 (1.8 µm, 2.1 mm*100 mm); column temperature, 40 °C; flow rate, 0.4 mL/min; injection volume, 2µL; solvent system, water (0.1% formic acid): acetonitrile (0.1% formic acid); gradient program, 95:5 V/V at 0 min, 10:90 V/V at 11.0 min, 10:90 V/V at 12.0 min, 95:5 V/V at 12.1 min, 95:5 V/V at 14.0 min.

(2) Amide UPLC Conditions: UPLC: column, Waters ACQUITY UPLC BEH Amide 1.7 µm,2.1 mm*100 mm; column temperature, 40 °C; flow rate, 0.4 mL/min; injection volume, 2µL; solvent system, water (25mM Ammonium formate/0.4% Ammonia): acetonitrile; gradient program, 10:90 V/V at 0 min, 40:60 V/V at 9.0 min, 60:40 V/V at 10.0 min, 60:40 V/V at 11.0 min, 10:90 V/V at 11.1 min, 10:90 V/V at 15.0 min.

(3) ESI-QTRAP-MS/MS: T3 and Amide have the same mass spectrometry parameters. LIT and triple quadrupole (QQQ) scans were acquired on a triple quadrupole-linear ion trap mass spectrometer (QTRAP), QTRAP® LC-MS/MS System, equipped with an ESI Turbo Ion-Spray interface, operating in positive and negative ion mode and controlled by Analyst 1.6.3 software (Sciex). The ESI source operation parameters were as follows: source temperature 500 °C; ion spray voltage (IS) 5500 V (positive), -4500 V (negative); ion source gas I (GSI), gas II (GSII), curtain gas (CUR) was set at 55, 60, and 25.0 psi, respectively; the collision gas (CAD) was high. Instrument tuning and mass calibration were performed with 10 and 100 µmol/L polypropylene glycol solutions in QQQ and LIT modes, respectively. A specific set of MRM transitions were monitored for each period according to the metabolites eluted within this period.

Quality control analysis

Quality control (QC) samples were prepared from the extract mixture of all test samples and analyzed under the same conditions to monitor test reproducibility. During the sample detection process, QC samples were analyzed every 10 samples to ensure the stability of the analysis process. First, we performed overlapping display analysis on total ion current (TIC) of mass spectrometry monitoring and analysis of different quality control samples to judge the repeatability of metabolite extraction and detection. The curve overlap of metabolite TIC was high, which means that the retention time and peak intensity were consistent, indicating that the signal stability of mass spectrometry was good when the same sample was detected at different times (Figure S2a, b). Figure S2C showed the Coefficient of Variation distribution of QC samples. The higher the proportion of substances with lower CV values in QC samples, the more stable the experimental data. The proportion of substances with CV value < 0.5 in QC samples was more than 85%, indicating that the experimental data was relatively stable,

while the proportion of substances with CV values < 0.3 in QC samples was higher than 75%, indicating that the experimental data was very stable. Finally, Pearson analysis was performed to evaluate the correlation of QC samples. The higher the correlation (|r| is closer to 1), the better the stability of the whole detection process and the higher the data quality (Figure S2d).

Supplementary Figures

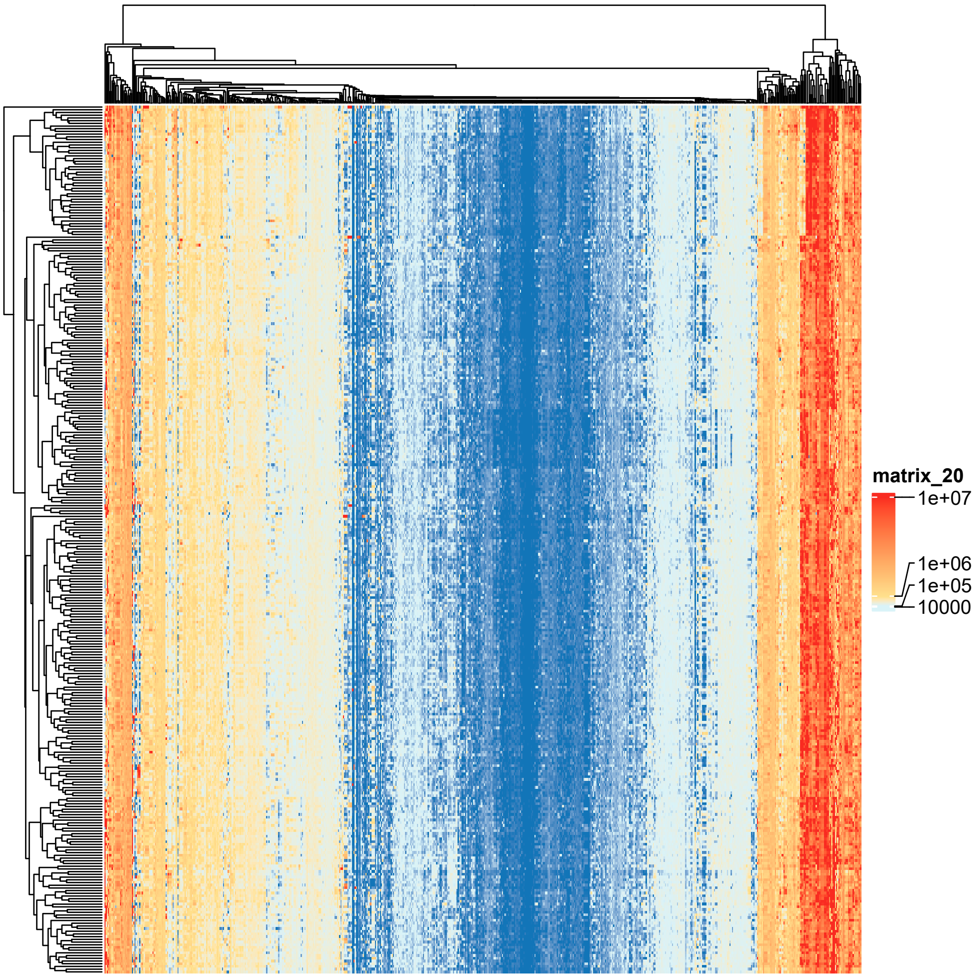

Figure S1. Heatmap visualization of 746 identified metabolites

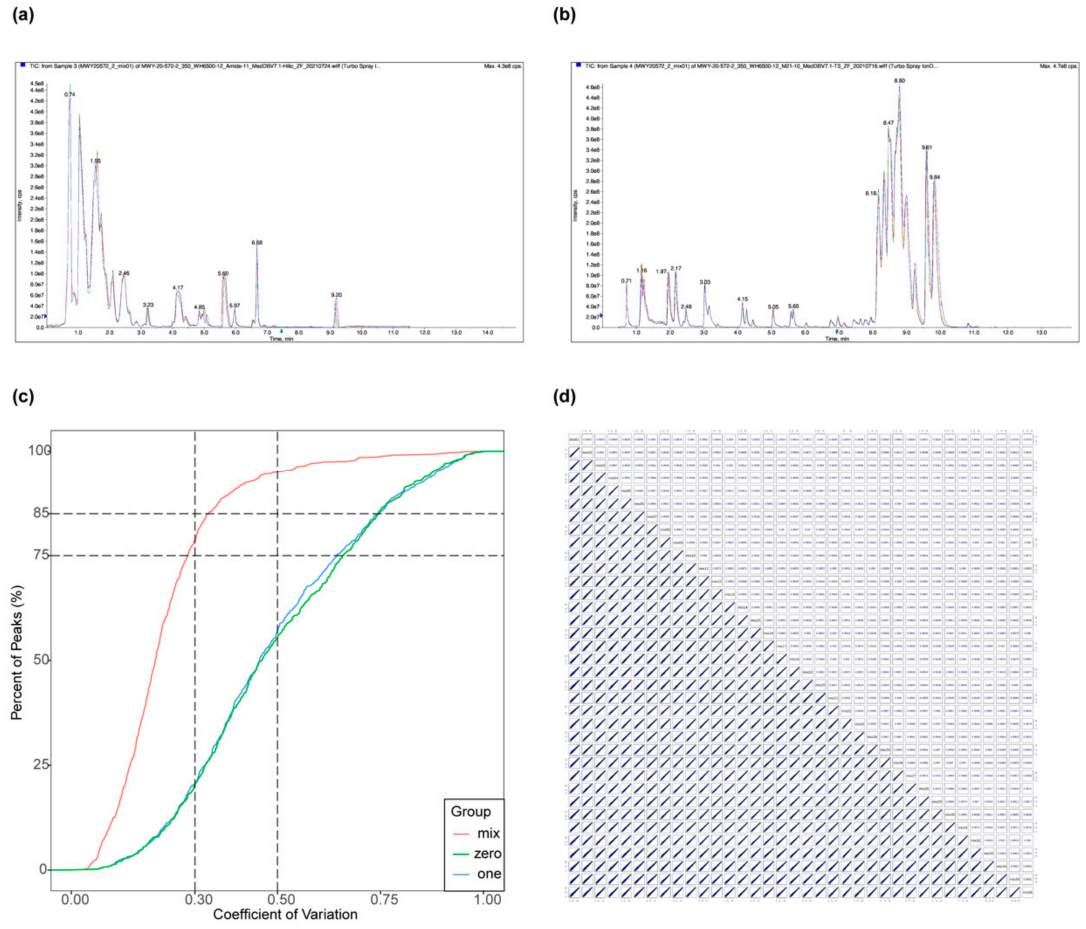

Figure S2. Sample quality control analysis. (a) Total ion chromatogram for mass spectrometry analysis of polyculture samples. QC_MS_Hilic-TIC. (b) Total ion chromatogram for mass spectrometry analysis of polyculture samples. QC_MS_T3-TIC. (c) CV distribution of QC samples. The abscissa represents the CV value, and the ordinate is a smaller number than the corresponding material of the CV value, accounting for the proportion of the total number of materials. Two references of the X-axis vertical line corresponding to CV values of 0.3 and 0.5, parallel to the X-axes of two reference lines corresponds to the material number, accounting for 75% and 85% of the total number of substances, respectively. (d) the Pearson correlation of QC samples. The higher the correlation (|r| is closer to 1), the better the stability of the whole detection process and the higher the data quality. The diagonal grid represents the name of the QC sample; the lower left corner of the diagonal grid is the corresponding QC sample correlation scatter diagram, the horizontal and vertical coordinates are the metabolite content (log processing), and each point in the figure represents a metabolite; The upper right corner of the diagonal is the Pearson correlation coefficient of the corresponding QC sample.

QC: Quality controls； TIC: Total ions current； CV: Coefficient of variation.

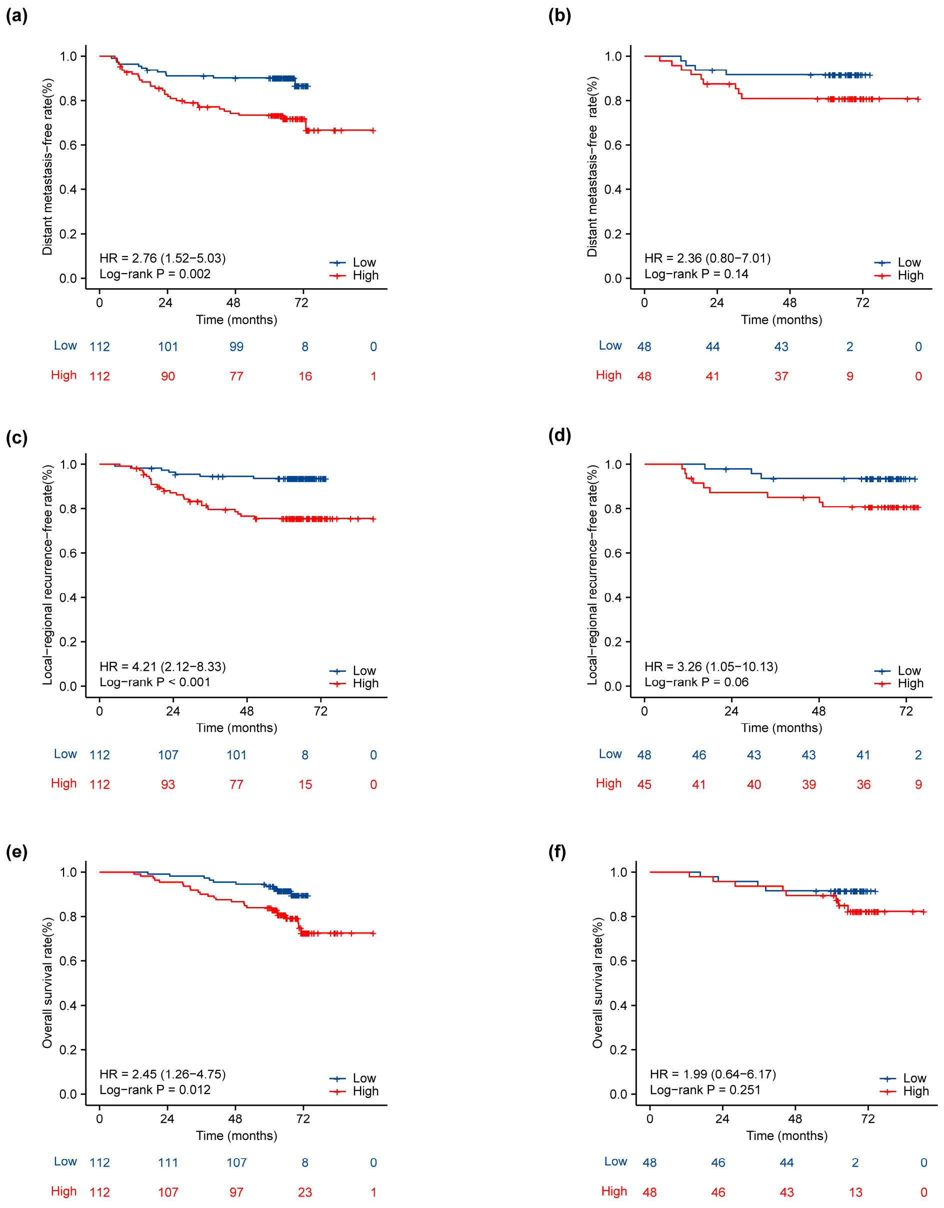

Figure S3. The Kaplan - Meier curves of the 9-metabolite signature for predicting secondary endpoints in training and validation sets. (a) Kaplan–Meier curves for DMFS in the training set (b) Kaplan–Meier curves for DMFS in the validation set. (c) Kaplan–Meier curves for LRFS in the training set. (d) Kaplan–Meier curves for LRFS in the validation set. (e) Kaplan–Meier curves for OS in the training set. (f) Kaplan–Meier curves for OS in the validation set.

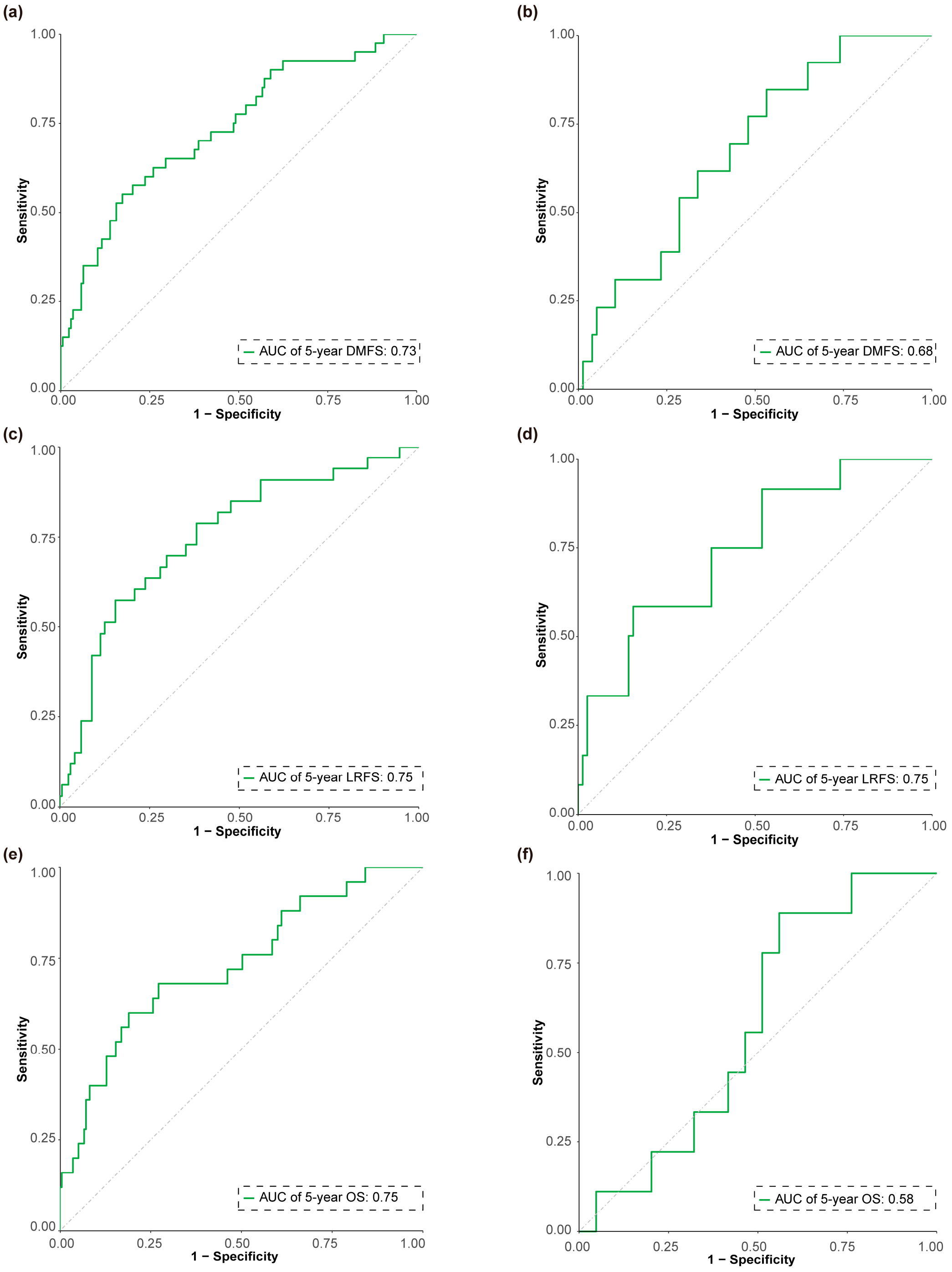

Figure S4. Receiver operating characteristic curve of the 9-metabolite signature for predicting secondary endpoints in training and validation sets. (a) ROC curve for DMFS in the training set. (b) ROC curve for DMFS in the validation set. (c) ROC curve for LRFS in the training set (d) ROC curve for LRFS in the validation set. (e) ROC curve for OS in the training set. (f) ROC curve for OS in the validation set.

Supplementary Tables

Table S1 is displayed as an excel file.

Table S2. The information of 27 metabolites associated with PFS by univariate Cox regression

NUMBER	ID	Compounds	P	HR	CI_low_0.95	CI_up_0.95	FDR
1	MEDN0554	2-(4-Hydroxyphenyl) ethanol	8.35E-09	1.408	1.253	1.582	6.23E-06
2	MADP0090	4-Guanidinobutyric Acid	1.24E-05	1.429	1.217	1.676	0.005
3	MADP0391	Pyr-Glu	2.82E-05	1.330	1.164	1.520	0.006
4	MEDP1294	Leu-Gly	3.84E-05	0.473	0.331	0.675	0.006
5	MEDN1224	Bis(1-inositol) -3,1'-phosphate 1-phosphate	3.87E-05	1.327	1.160	1.518	0.006
6	MEDN1666	N-Acetyl-L-Glutamic Acid	4.91E-05	1.339	1.163	1.542	0.006
7	MEDP0087	L-Alanyl-L-Lysine	5.18E-05	1.340	1.163	1.545	0.006
8	MADP0394	Ile-Ser	<0.001	1.378	1.171	1.622	0.011
9	MEDN1406	Tridecanedioic acid	<0.001	1.420	1.186	1.700	0.011
10	MEDP1894	Tyr-Leu	<0.001	0.534	0.386	0.738	0.011
11	MADN0456	Ile-Phe	<0.001	0.428	0.273	0.671	0.015
12	MEDP1234	Dopaquinone	<0.001	1.229	1.101	1.373	0.015
13	MADP0344	5-methylcytidine	<0.001	1.350	1.149	1.586	0.015
14	MEDP1506	Leu-Val	<0.001	0.533	0.372	0.762	0.029
15	MEDN1475	Sorbitol 6-phosphate	<0.001	1.334	1.132	1.573	0.029
16	MADP0384	Val-Leu	<0.001	0.509	0.345	0.749	0.029
17	MEDP1889	Ser-Phe	<0.001	0.560	0.399	0.785	0.031
18	MEDP1962	N-(3-Indolylacetyl)-L-alanine	<0.001	1.354	1.134	1.616	0.031
19	MADN0457	Ile-Val	<0.001	0.582	0.425	0.799	0.031
20	MEDP1710	11-Ketoetiocholanolone	<0.001	1.381	1.142	1.671	0.031
21	MEDP1098	Methoxyindoleacetic Acid	<0.001	1.340	1.127	1.593	0.031
22	MEDP1916	N-Acetyl-L-phenylalanine	<0.001	1.364	1.136	1.640	0.031
23	MADP0177	N-Formylglycine	0.001	1.341	1.124	1.599	0.036
24	MADP0429	Val-Ile	0.001	0.513	0.341	0.770	0.040
25	MADP0036	Gly-Leu	0.001	1.279	1.100	1.488	0.043
26	MEDN0745	Propylparaben	0.002	1.308	1.108	1.545	0.044
27	MEDN1706	3-(2-Hydroxyphenyl) propanoic acid	0.002	1.388	1.131	1.703	0.047
